# Entropy in the Assessment of the Labour Market Situation in the Context of the Survival Analysis Methods

**DOI:** 10.3390/e27070665

**Published:** 2025-06-21

**Authors:** Beata Bieszk-Stolorz

**Affiliations:** Institute of Economics and Finance, University of Szczecin, 71-101 Szczecin, Poland; beata.bieszk-stolorz@usz.edu.pl

**Keywords:** entropy, survival analysis, unemployment duration, unemployment rate

## Abstract

Since Shannon’s pioneering work, the concept of entropy has been used in many major scientific fields. It is therefore a universal concept but also defined in different ways. Entropy is used in studies of system complexity and to investigate the information content of probability distributions. One of the areas of its applications is human lifespan, i.e., the link between entropy and the methods of survival analysis. These methods are also used in assessing the duration of any socio-economic phenomenon. The aim of this article is to assess the market situation on the basis of the entropy of duration in unemployment. This study determines the Shannon entropy, residual entropy, past entropy, and cumulative residual entropy under the assumption of an exponential distribution of duration. Ward’s hierarchical clustering and the Dynamic Time Warping measure were used to analyse entropy and its relationship with the unemployment rate. It was shown that not all of the analysed models determine the entropy of duration in unemployment well for an exponential distribution. It was substantiated that there is a similarity between the formation of the entropy of duration in unemployment and the registered unemployment rate. It is shown that high unemployment rates in the labour market are a destabilising element of the labour market, more so than crises.

## 1. Introduction

The concept of entropy in its original version was developed in the field of thermodynamics and was later extended to statistical mechanics [1]. Since Shannon’s [2,3] pioneering work on the mathematical theory of communication, entropy has been used as a major tool in engineering and information theory. The concept is also fundamental in other scientific fields such as probability and statistics, demography, economics, finance, and actuarial sciences [4,5,6,7]. Entropy is used in studies of system complexity and to investigate the information content of probability distributions. As entropy is a general measure, many applications and definitions have been proposed in the literature [8]. One of the areas of application of this concept is the analysis of the duration of human life [1,9,10]. Among the indicators of uncertainty associated with survival studies are Wiener’s entropy index [11], Shannon’s lifetime entropy index (Meyer’s entropy) [9], and Hill’s entropy index [12]. However, survival analysis does not only focus on the duration of human life. It can also refer to a random variable that describes the duration of any socio-economic phenomenon. This can be the duration of unemployment [13,14], the duration of firms [15], the duration of civil wars, post-war peace, and alliances [16,17,18], and the probability and intensity of decline and increase in stock prices in the capital market [19]. Survival analysis methods can also be used in the analysis of other socio-economic phenomena: population economic activity [20], poverty dynamics [21], credit risk [22], and the real estate market [23]. Many studies related to the entropy of human lifespan have appeared in the literature. In contrast, there is a lack of research related to the entropy of the duration of socio-economic phenomena. The analysis presented in the article fills this research gap. This is because the conducted study concerns the possibility of applying entropy in the analysis of duration in unemployment, which, next to the unemployment rate, is one of the most important elements in the assessment of the labour market. Survival analysis methods were first used in reliability theory and demography. They have also been successfully used in socio-economic research for many years. Due to the form of the data (unit data and individual data), they usually cover a short research period. It often happens that the research sample is not fully representative (from a statistical point of view). The present study is unique due to the following three facts: Firstly, it covers a long research period—15 years. Secondly, it covers the entire population of people registered with the Poviat Labour Office in Szczecin (Poland). This is a total of 269,872 people. Thirdly, to my knowledge, there are no examples in the literature of the use of entropy in labour market analysis using survival and hazard functions.

The aim of this article is to assess the market situation on the basis of the entropy of duration in unemployment. In this study, individual data obtained from the District Employment Office in Szczecin (Poland) were used. The research period was 2007–2021. This period includes the global financial crisis in 2007–2009 [24] and the outbreak of the COVID-19 pandemic in 2020–2021 [25,26]. Survival models were constructed assuming an exponential distribution of duration in unemployment separately for each of the analysed years. Some unemployed persons were de-registered from the office for reasons other than undertaking work. Such observations constituted right-censored observations. Survival time models allow such observations to be included in this study.

An analysis of the available studies on the entropy of the duration of human life suggests that such models can be applied to the analysis of the duration of any socio-economic phenomenon. However, when drawing conclusions, it should be remembered that in the case of human life, the event that ends the observation is the death of the observed individual. This is a negative event. The longer the time to this event, the better. In the case of the analysis of the duration of unemployment, the event that ends the observation is undertaking a job, i.e., a positive event. In such a case, the shorter the time, the better. In this study, the following two research questions were formulated:

Q1: Do the analysed models determine the entropy of duration in unemployment well, assuming an exponential distribution?

Q2: Is there a similarity between the development of the entropy of duration in unemployment and the registered unemployment rate?

The rest of the article is organised as follows: Chapter 2 presents the concept of entropy in the context of survival analysis, assuming an exponential distribution of duration. Chapter 3 presents the data used and the research methods. Chapter 4 presents the results of the empirical analysis. Chapter 5 presents a discussion of the obtained results in the context of similar studies. Chapter 6 is a summary of the entire research.

## 2. Entropy in the Survival Analysis

The concept of entropy is particularly important in the field of information theory and was introduced by Claude E. Shannon in 1948 [2,3]. The original definition was given for discrete random variables. Let *X* be a finite, discrete random variable for which the probability mass function is defined as $p_{i}=P(X=x_{i})$, for i=1,…,n, where $\sum_{i=1}^{n}p_{i}=1$ and $p_{i}\geq 0$. The Shannon entropy *H* of the random variable *X* is defined as follows [2]:

(1) $$H=-\sum_{i=1}^{n}p_{i}\ln p_{i}dx.$$

Expression (1) has been extended to the continuous case. If *X* is a random variable with a continuous distribution with probability density function *f*, then the Shannon entropy *H* is given by the following formula [3]:

(2) $$H=-\int_{-\infty }^{\infty }f\left(x\right)\ln f\left(x\right)dx.$$

It is commonly referred to as the Shannon measure of information. Because of the assumption of continuity of the density function *f*, this entropy is also called the differential entropy or continuous entropy [27].

Let us consider a continuous non-negative random variable *X* with a probability density function *f*. Let f(x)=0, for x≤0. Then, the Formula (1) takes the following form:

(3) $$H=-\int_{0}^{\infty }f\left(x\right)\ln f\left(x\right)dx$$

Formula (3) represents a measure of uncertainty for *X*.

Entropy *H* represents the expected uncertainty contained in *f* regarding the predictability of the value of a random variable *X* [28,29]. This means that entropy measures the concentration of probabilities. Low-entropy distributions are more concentrated, hence more informative, than high-entropy distributions.

### 2.1. Basic Functions in the Survival Analysis

Survival (duration) analysis methods are used when the duration of a given phenomenon is studied and it is described by a random variable *T*. Duration analysis uses individual data on each individual studied. This is quite a complication, as most of the data are given in aggregate form. However, the advantage of such an analysis is that incomplete (censored) data can be used. Right-censored data influence the shape of the survival curve $S\left(t\right)$. Survival curves are estimated taking such data into account. The parameters of the distribution are determined on the basis of the survival curves. Next, entropy is calculated on the basis of the estimators of these parameters.

The basic function in survival analysis is the survival (duration) function *S* described by the following formula [30]:

(4) $$S\left(t\right)=1-F\left(t\right)=P\left(T>t\right)=\int_{t}^{\infty }f\left(u\right)du,$$

where:

$f\left(t\right)$—probability density function of random variable *T*.
$F\left(t\right)$—cumulative distribution function.

The survival function defines the unconditional probability that the event of interest has not occurred until the moment *t* [31].

The second important function is the hazard function *h*, describing the intensity of the event at time *t* [30]:

(5) $$h\left(t\right)=\underset{∆t\to 0}{\lim}\frac{P\left(t\leq T<t+∆t\left|T\geq t\right.\right)}{∆t}.$$

There are several relationships between the functions *f*, *S* and *h*. The most important ones are as follows:

(6) $$h\left(t\right)=\frac{f\left(t\right)}{S\left(t\right)},$$

(7) $$h\left(t\right)=-\frac{S^{\prime }\left(t\right)}{S\left(t\right)}.$$

There are various approaches used in research related to the distribution of survival time. There are non-parametric models (Kaplan–Meier estimator [32] and Nelson–Aalen estimator [33,34,35]), semiparametric models (Cox hazards model [36]), and parametric models based on different assumptions about the form of the duration function.

### 2.2. Entropy in the Assessment of Uncertainty of Life Expectancy

Let *T* be a non-negative random variable with the cumulative probability distribution F(t)=P(T≤t). We then define the survival function *S* as follows: S(t)=P(T>t), where S(0)=1. The random variable *T* can be interpreted as the failure time of a component or system, the human lifetime or the duration of an event. If *T* represents the lifetime of an individual, then entropy *H* can be used as a potential measure of the associated uncertainty. The amount of information obtained after the disclosure of age at death can be measured by the entropy of life. Entropy is a general measure of the information revealed by an event [37]. It can be thought of as a measure of resolved uncertainty. When we apply it to uncertainty about life expectancy, it allows us to measure the degree of risk associated with age at death. The event under study is therefore the “death of someone”. The information we learn from the event “death at a given age” is given by the measured entropy of life [9]. In the case of human life, Shannon’s lifetime entropy index allows humans to become more familiar with the risk about the duration of life. During early adulthood, the entropy of life is high, as there can be a large number of life expectancy scenarios. When an individual reaches old age, the number of possible life expectancy scenarios becomes smaller and the entropy of human life decreases. The calculation of Shannon’s lifetime entropy indices over successive lifetimes informs how the amount of life expectancy risk changes with age as individuals become older [10]. Lifetime entropy can be considered the informative equivalent of standard life expectancy. Instead of measuring mathematical life expectancy, entropy measures the expected quantity of information revealed by a particular duration of life. The amount of information revealed by the death of someone depends on the following [10]:

- The age at which the death occurred.
- Distribution of possible life expectancies at the age of death.

Studying the impact of age-specific mortality on lifetime entropy is important for the following reasons [10]:

1. When there is an age-related change in the probability of death, this change affects the entire life expectancy distribution. Some lifetimes become more likely and others less likely than before the change. The net effect on the lifetime entropy is difficult to estimate.
2. Individual decisions on, e.g., insurance depend on life expectancy and the magnitude of life expectancy risk. A question arises—how do mortality shocks (for instance, epidemics) affect life expectancy and lifetime entropy?
3. In the world’s economies, mortality shocks may affect various life periods differently. It is therefore important to examine how the relationship between lifetime entropy and mortality shocks changes with age.

An interesting and important proposal for using entropy in survival analysis is the entropy of the population [38]. With this measure, it is possible to measure how relative changes in the mortality function affect the relative change in the life expectancy of the population [39]. This entropy is also called the life table entropy, also known as the Keyfitz entropy [40].

If an individual has survived to age *t*, then information about the remaining age is of particular importance in reliability and survival analysis [41]. In his work, Ebrahimi [42] stated that if an individual is known to have survived to age *t*, then *H* is no longer useful for measuring uncertainty about the remaining lifetime of the individual. He has therefore introduced a measure of uncertainty in the residual lifetime distribution $H\left(T,t\right)$ estimated by means of the following formula:

(8) $$H\left(T,t\right)=-\int_{t}^{\infty }\frac{f\left(u\right)}{S\left(t\right)}\ln\frac{f\left(u\right)}{S\left(t\right)}du=1-\frac{1}{S\left(t\right)}\int_{t}^{\infty }f\left(u\right)\ln\frac{f\left(u\right)}{S\left(u\right)}du,$$

where:

$S\left(t\right)$—survival function.
$f\left(t\right)$—probability density.

Residual entropy of random variable *T* measures the uncertainty about its continuing lifetime under the assumption that a unit has survived up to time *t* [43]. This measure is therefore useful for comparing the residual lifetimes of two objects that have survived to time *t* [44].

In many real-life situations, uncertainty is not necessarily related to the future but can also refer to the past. It may happen that a system, which is observed only at certain predetermined moments, already turns out to be faulty before time *t* has elapsed. Then, the duration risk of the system depends on the past, i.e., at which point in time (0, *t*) the failure occurred [45]. Following this idea, Di Crescenzo and Longobardi studied the past entropy at time (0, *t*). If *T* denotes the lifetime of a system or living organism, then the past entropy $\bar{H}\left(T,t\right)$ is defined as follows [46]:

(9) $$\bar{H}\left(T,t\right)=-\int_{0}^{t}\frac{f\left(u\right)}{F\left(t\right)}\ln\frac{f\left(u\right)}{F\left(t\right)}du=1-\frac{1}{F\left(t\right)}\int_{0}^{t}f\left(u\right)\ln\frac{f\left(u\right)}{F\left(u\right)}du$$

where:

$F\left(t\right)$—the cumulative distribution function;
$f\left(t\right)$—probability density;
$\frac{f\left(t\right)}{F\left(t\right)}$—the reversed failure rate of *T* or reversed hazard function.

The reversed hazard function is receiving increasing attention in reliability theory and survival analysis [47,48].

Assuming that an event occurred at time *t*, $\bar{H}\left(T,t\right)$ measures the uncertainty of past life and uniquely determines the distribution of lifetimes [49]. Past entropy can also be considered as the entropy of idle time [50].

It is known that for t=0, we have $S\left(t\right)=1$ and $F\left(t\right)=0$. It follows that for t=0, residual entropy is equal to the differential entropy H [51]. In contrast, in this case there is no past entropy (which is consistent with the interpretation of past entropy).

In the discrete case, the Shannon entropy is always non-negative and equal to zero if and only if the random variable is a certain event. The entropy of a continuous distribution can be negative for certain probability distributions [3,42]. This makes it useless as a measure of uncertainty [52]. This is one of the most significant drawbacks of this measure. In response to this, Rao et al. [53] and Wang et al. [54] defined a new measure of uncertainty and called it the cumulative residual entropy (CRE) [55,56]. The density function *f* in definition (2) given by Shannon has been replaced by the cumulative distribution function. The cumulative distribution function is more regular than the density function because the density is calculated as its derivative. In practice, the cumulative distribution function is more interesting. For example, if the random variable is the life span of a machine, it is more interesting whether the life span exceeds *t* rather than whether the life span equals *t*. Cumulative residual entropy is based on the survival function of a positive random variable and is given by the following formula:

(10) $$CRE=-\int_{0}^{t}S\left(t\right)\ln S\left(t\right)du,$$

where $S\left(t\right)$ is the survival function.

The cumulative residual entropy refers to the information content of the distribution of a random variable and therefore also provides a measure of uncertainty. This measure can be used for both continuous and discrete random variables [43]. This measure is valid in both the continuous and discrete domains and can be easily calculated from sample data. An important advantage of this measure is that CRE is always nonnegative.

### 2.3. Entropy for the Exponential Distribution of Survival Time

If the distribution of the duration of a phenomenon is known, parametric methods are preferred to other methods (non-parametric or semiparametric) in the survival analysis. The advantages of the parametric methods in this case include the following [57]:

- The distribution of survival time can be estimated.
- Estimated parameters provide clinically meaningful estimates of effect.
- Residuals can represent the difference between observed and estimated values of time.
- Full maximum likelihood can be used to estimate parameters.

Distributions that are well suited to survival data are, among others, the exponential, Weibull, gamma and log-normal distributions. The Gompertz distribution provides a convenient way to describe survival in humans and is often used in demography. In veterinary epidemiology, the most important parametric forms are the exponential and Weibull distributions [58,59]. The exponential distribution is particularly useful in survival analysis because it assumes a constant hazard rate over time. It is often a good approximation in certain contexts, such as the time to decay of a radioactive particle. It is characterised by simplicity and lack of memory, meaning that future life expectancy is the same regardless of current age. Unfortunately, because a constant risk rate is rarely found in human and animal survival studies, the applicability of the exponential distribution is rather limited in these cases [60]. The exponential model is often considered in studies of duration in unemployment and job search time [61,62,63].

An exponential distribution of duration was used in this study to model the duration of unemployment. The process of leaving unemployment is described by the survival curve *S*(*t*). It is a non-increasing curve and, in the case of a continuous distribution, similar to a decreasing exponential curve. In addition, in the case of people registered with labour offices, it is observed that many of them undertake work within the first few months of registration. The distribution of time from registration to undertaking work is strongly right-skewed. In many cases, it is an exponential distribution.

In the case of an exponential distribution of survival time, the density function is assumed to be of the following form:

(11) $$f\left(t\right)=\lambda e^{-\lambda t\;\;\;\;}\mathrm{dla}\;\lambda >0.$$

where λ is the rate parameter. The distribution is supported on the interval [0, ∞). In that case, the basic functions in the survival analysis take the forms of:

(12) $$F\left(t\right)=1-e^{-\lambda t},$$

(13) $$S\left(t\right)=e^{-\lambda t},$$

(14) $$h\left(t\right)=\lambda .$$

Using Formulas (3) and (11), the Shannon entropy for an exponential distribution can be determined. It is expressed by the following formula:

(15) H=1−lnλ.

In the case of the exponential distribution, the uncertainty of the residual lifetime distribution is given by the following formula [64]:

(16) $$H\left(T,t\right)=1-\ln\lambda$$

For an exponential distribution, we therefore have: $H=H\left(T,t\right)$.

In contrast, the past entropy determined by the Formulas (9), (11), and (12) is of the following form [64]:

(17) $$\bar{H}\left(T,t\right)=\ln\frac{1-e^{-\lambda t}}{\lambda }+1-\frac{\lambda te^{-\lambda t}}{1-e^{-\lambda t}}.$$

For the exponential distribution (Formulas (10) and (13)), the cumulative residual entropy is equal [65].

(18) $$CRE=\frac{1}{\lambda }$$

From the mentioned formulas it can be observed that for an exponential distribution the entropy *H*, residual entropy, and CRE are constant. Only the past entropy depends on time. In addition, the following properties are true:

(19) $$\underset{t\to 0^{+}}{\lim}\bar{H}\left(T,t\right)=-\infty$$

and

(20) $$\underset{t\to \infty }{\lim}\bar{H}\left(T,t\right)=1-\ln\lambda .$$

It follows from the Formulas (15) and (16) that in the case of the exponential distribution for λ>e (e—Euler’s number), entropy *H* and residual entropy are negative. Past entropy is negative for very small values of *t* (Formula (19)). Negative entropy values are one of the drawbacks of the Shannon entropy measure for a continuous random variable. On the other hand, the cumulative residual entropy, denoted by Formula (18), is always positive (because λ>0) and it is a better measure of entropy in the case of the exponential distribution.

## 3. Data and Research Methodology

In this research, data on unemployed persons de-registered from the Poviat Labour Office in Szczecin (Poland) between 2007 and 2021 have been used. These are the individual data collected in their teleinformatic system. They contain the date of registration, the date of de-registration and information on the reason for de-registration. The random variable *T* describes the length of time an unemployed person was registered with the office. The event that ends the observation of a registered individual is undertaking work. De-registration for a reason other than undertaking work (inter alia: retirement, going abroad, continuing education, and resignation from the intermediation of the office) is a right-censored observation. Table 1 provides information on the size of the groups of unemployed persons. In addition, the average length of time registered in total and until undertaking work was determined. These values were compared with the unemployment rate in Poland. The numerical values in Table 1 indicate a high percentage of people de-registered for reasons other than undertaking work. Analysis of the primary data showed that this was influenced by the percentage of people who were de-registered for reasons that were their own fault. In general, it can be concluded that these were persons who gave up their co-operation with the office.

In studies using survival analysis methods, the main problem is access to data. In the methods presented, the data must be individual, not aggregate. Therefore, the data used in this study are unique. They are individual data and concern each person registered with the labour office. The data come from the new version of their teleinformatic system. Before 2007, the another system was used in labour offices in Poland. Of course, data from the old system were transferred to the new one, but this resulted in significant gaps in the data and some inaccuracies. That is why it was decided to start this study in 2007. The completion of the survey for 2021 is due to the fact that it was possible to obtain data only up to this year.

**Figure 1:**
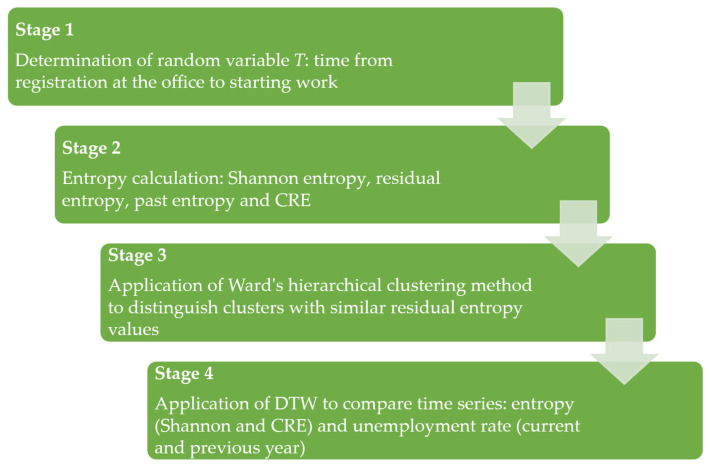
Research scheme.

The survey was conducted in four stages (Figure 1). In the first stage of this study, a random variable *T* was determined for each analysed year (2007–2021), which describes the time from registration to de-registration from the office. Undertaking work is a full observation, which was coded as 1. De-registration due to other reasons (censored observation) was coded as 0. In the second stage of this study, Shannon entropy, residual, past entropy and CRE were determined. The third stage consisted of using hierarchical clustering (with using the Ward’s method) to isolate clusters with similar values of residual entropy. In the fourth stage, the Dynamic Time Warping (DTW) method was used to compare the time series: entropy (Shannon and CRE) and unemployment rate (current and past year).

To compare the time series for determined entropy and unemployment rates, Dynamic Time Warping (DTW) was used. It was developed by Bellman and Kalaba [66] to address the speech recognition problems [67,68,69,70]. It is currently used in many research areas, e.g., in the field of music information retrieval [71], in bioinformatics [72], for gesture recognition [73], in finance [74], for commodity price analyses [75] and in the labour market [76]. It is used to determine the optimal match between two time series by stretching, compressing, or stretching them locally so that one is as similar as possible to the other. This distortion (called warping) allows the time axis to be adjusted to find similar but phase-shifted sequences [77].

## 4. Results

Stage one of this study was carried out while compiling the data received from the labour office. In addition, the average time of registration overall and until employment was determined (Table 1). The results of the second stage of the research are presented in Table 2. It contains the results of estimating the parameter λ for the exponential distribution of duration in unemployment. Using Formulas (15) and (16), the Shannon entropy and the residual entropy were also determined. Obviously, for an exponential distribution, the values of both entropies are the same. For all analysed years, the parameter λ<e results in positive values of the Shannon entropy and residual entropy, i.e., they are well-defined measures.

An analysis of Formulas (15) and (18) indicates that decreasing values of the parameter λ correspond to increasing values of the Shannon entropy and the cumulative residual entropy, and vice versa. This is reflected in the values contained in Table 2. This is related to the fact that for an exponential distribution of duration, higher values of the parameter λ correspond to lower values of duration. The higher the value of the parameter λ, the faster the survival curve decreases. This means that unemployed people find work faster. However, there is no analogous regularity in the case of the λ parameter and unemployment rates.

**Figure 2:**
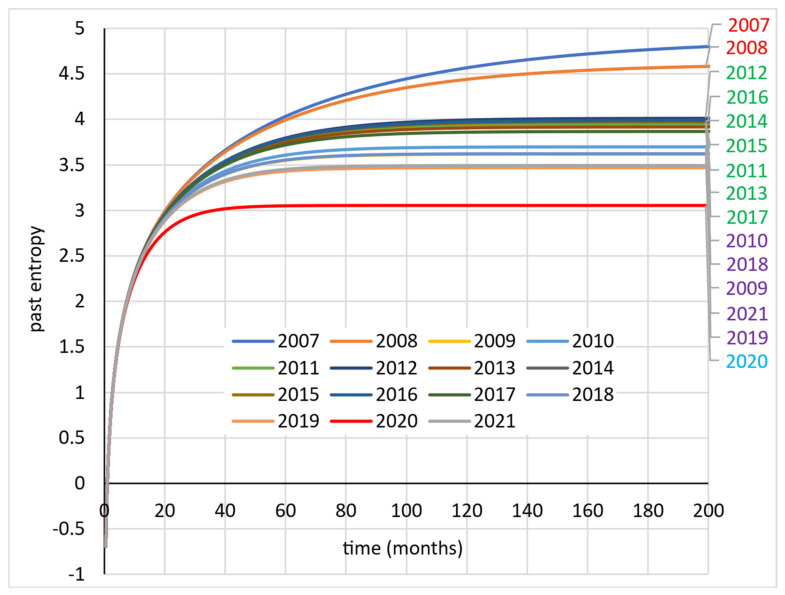
Past entropy for the exponential distribution of duration in unemployment.

Figure 2 shows graphs of past entropy as a function of duration in unemployment *t*. All graphs as *t* increases approach the corresponding Shannon entropy values, which is consistent with Equation (20). As there is little variation in the 1−lnλ values, the analysed years are listed on the right-hand side of the graph in the appropriate order.

Unfortunately, the main disadvantage of the Shannon entropy is the possibility of negative values for different duration distributions. A negative value may appear at the stage of calculating the Shannon entropy, residual entropy or past entropy. In this study, a negative value appeared at the stage of calculating past entropy. In all cases, the past entropy for a short time *t* (t≤1) takes values less than zero. In the database used, there are individuals who enter employment very quickly (in time equal to or less than one month). Therefore, the situation that $\bar{H}\left(T,t\right)<0$ is by all means probable. Therefore, in the subsequent stages of the research, the cumulative residual entropy was determined from Equations (10) and (18) (Table 1). This measure is always positive. CRE takes higher values than the other entropy measures used in the article. However, the direction of change from 2007 to 2021 is the same.

**Figure 3:**
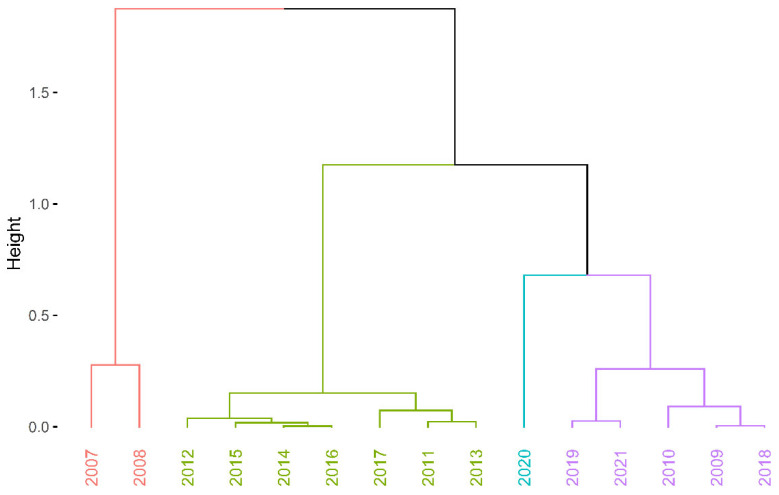
Results of hierarchical clustering by using the Ward’s method.

The considerations outlined above provide an answer to question Q1. Not all the considered models determine the entropy of duration in unemployment accurately. Residual entropy and CRE are positive, so they can be fully used to assess the labour market situation in Szczecin between 2007 and 2021. Past entropy, on the other hand, is unsuitable for assessing duration in unemployment in the case where it is short. However, the course of the curves in Figure 2 indicates that the curves can be clustered according to boundary values, i.e., residual entropy values. This is the third stage of this study. The Ward’s hierarchical clustering method [78] was used for this purpose. The distance matrix between entropy values in individual years was determined by using the Euclidean metric. The optimal number of clusters was determined in the R environment [79] with the use of the NbClust package [80]. The largest number of indices indicated four as the optimal number of clusters. The result of this clustering is shown in Figure 3. Four clusters of years were obtained, of which three were continuous periods and one contained years that did not form a continuous sub-period:

- Cluster 1—years 2007–2008—high entropy.
- Cluster 2—years 2011–2016—medium high entropy.
- Cluster 3—years 2009–2010, 2017–2019, and 2021—medium low entropy.
- Cluster 4—year 2020—low entropy.

The colours of these groups in Figure 3 are the same as the font colours used to write the years in Figure 2. Cluster 1 and Cluster 4 contain outlier observations. Both clusters refer to labour market shocks. Cluster 1 is the beginning (2007–2008) of the global financial crisis in 2007–2009. Entropy in these years is high. Cluster 4 is only the year 2020 and therefore the beginning of the COVID-19 pandemic. In this year, the entropy was low. The remaining years are either in Cluster 2 or Cluster 3. They are characterised by entropy with average values, with Cluster 2 having slightly higher values than Cluster 3. It can therefore be concluded that during periods of labour market shocks, entropy (Shannon entropy, residual entropy and CRE) took on extreme values.

**Figure 4:**
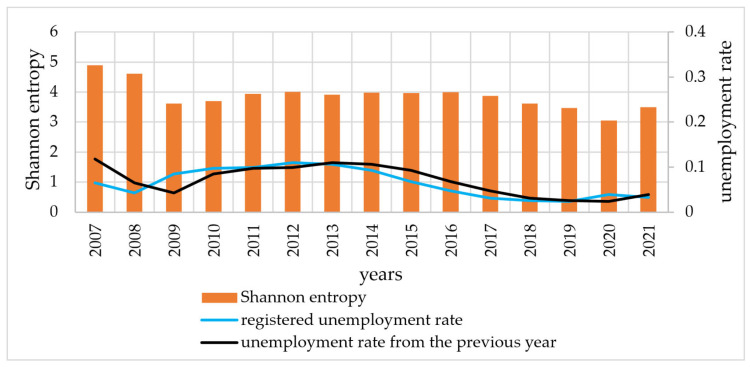
Shannon entropy values and unemployment rates in 2007–2021.

**Figure 5:**
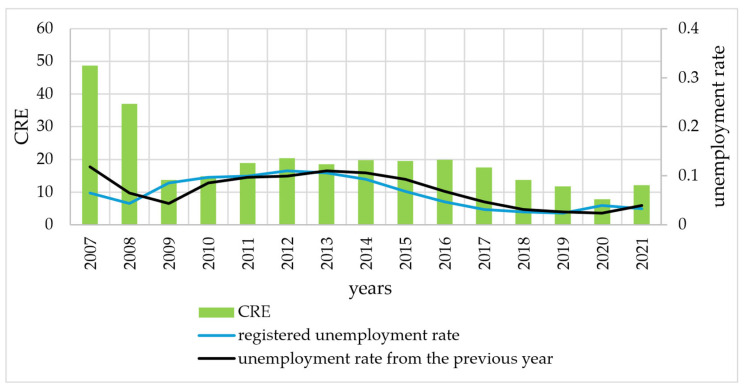
Values of CRE and unemployment rate in 2007–2021.

There was therefore no clear pattern in the entropy values in relation to the occurrence of a crisis situation. Therefore, the fourth stage of this study juxtaposes entropy values with unemployment rates. This stage is intended to provide an answer to research question Q2. The Shannon entropy (Figure 4) and CRE (Figure 5) were juxtaposed with the registered unemployment rate in Szczecin. Analysis of the graphs confirms the conjecture of a relationship between the unemployment rate and entropy. In order to accurately assess this relationship, in Figure 4 and Figure 5, unemployment rate graphs shifted back one year (unemployment rate from the previous year).

A preliminary analysis of the graphs indicates that changes in entropy (Shannon and CRE) follow a similar pattern to changes in unemployment rates. In the case of the previous year’s unemployment rate, the similarity is more pronounced. To confirm these observations, the DTW distance was used in further analysis. Table 3 presents the DTW distances, median and mean differences (in years) between the entropy (Shannon and CRE) and unemployment rates. The DTW distances are smaller for the Shannon entropy, indicating that changes in the Shannon entropy are more similar to changes in the unemployment rate than for changes in the CRE. On the other hand, shifting the unemployment rate by 1 year causes the similarity to increase for both entropy measures. This fact is confirmed by the determination of both median and mean shifts. This study stage provides an affirmative answer to research question Q2. There is a similarity between the development of the entropy of duration in unemployment and the registered unemployment rate. High values of the unemployment rate correspond to high values of entropy and vice versa. It follows that in periods of high unemployment, the distributions of duration in unemployment are less informative than in periods of low unemployment (measured by the level of the unemployment rate). If we take entropy as a measure of disorder, it can be concluded that high unemployment rates in the labour market are a destabilising element of the labour market, more so than crises. This is evidenced by the situation in 2020. This is the first year of the COVID-19 pandemic in which the designated entropy was the lowest in the period analysed. At the same time, there was a relatively low unemployment rate in that year.

## 5. Discussion

Studies on the magnitude of entropy in the face of crises have appeared in the literature. These are mainly articles on developments in financial markets. The results of these studies have not always been conclusive. Oh et al. [81] analysed entropy values of the four periods for three markets, i.e., the DAX, S&P 500, and KOSPI indices. For the DAX market index, there were no drastic changes, and the entropy volatility was low. The entropy value of the S&P 500 index fell until the subprime crisis (July 2007–December 2008) and then rose sharply after the financial crisis. The entropy value of the KOSPI market showed a significant increase after the US subprime crisis compared to the earlier bull market (July 2004–June 2007).

Anagnoste and Caraiani [82] investigated how macroeconomic and financial variables affected the entropy measure of financial markets. They showed that entropy responded positively to monetary policy shocks, although the effect was not statistically significant. In contrast, they found positive and statistically significant entropy responses to shocks in industrial production and the DJIA index.

Hou et al. [83] analysed the temporal variation of permutation entropy (PE) in Chinese stock markets. They investigated whether the complexity or degree of information availability changed during market crashes (the complexity or degree of information varies during market crashes). They observed that PE declined significantly in two significant periods—each encompassing a rapid market rise and then a few gigantic stock crashes.

Caraiani [84] showed that measures of entropy had the potential to reveal the state of markets. In each case analysed, the entropy measure fell just before the 2007-08 financial crisis, while it rose sharply after the onset of the crisis.

Wang and Wang [85] evaluated the time-varying informational efficiency through the refined composite multiscale fuzzy entropy (RCMFE) method. The authors assessed the market efficiency of the S&P 500 index, gold, bitcoin, and the US dollar index during the COVID-19 pandemic. COVID-19 led to a decline in efficiency in all four markets. The decline was particularly large for the S&P 500 index. The continuous downtrend of the stock index triggered a downtrend that lowered entropy and efficiency.

Olbryś and Majewska [86] assessed and compared the regularity in changes in European and American stock indices during major turbulence periods. The authors verified the research hypothesis that the entropy of an equity market index decreased during turbulence periods was examined. The findings imply that regularity in stock market index returns increases during extreme event periods.

There have also been publications in the literature on the use of entropy in labour market analysis. Rodríguez and Cáceres-Hernández [87] hypothesised that information is the primary source of economic value. The higher the socio-economic information, the greater the deviation in human effort towards the preferred direction, and thus the greater the reduction in internal entropy. The main result of their analysis is consistent with Adam Smith’s view, who attributed the source of national wealth to the social division of labour. The researchers showed that the higher the socio-economic information, the higher the bias in human effort towards a favoured direction and, therefore, the higher the reduction in internal entropy.

Popkov et al. [88] modelled labour market dynamics. They constructed a model of employment structures between cohorts for nine EU countries, identified its parameters and examined its adequacy. Their research was based on the concept of a positive dynamic system with an entropy operator.

Entropy is used to study the discrepancy between the distributions of employed and unemployed people. The Kullback–Leibler relative entropy measure is suggested as a very practical way of measuring the equality of opportunities for young people in employment in relation to their educational achievements, as well as for measuring differences in the distributions of employed and unemployed people depending on their level of education [89].

The use of entropy in labour market analysis can lead to unexpected results. A study conducted by Attaran [90] showed that the use of entropy as a reliable economic indicator yields weaker results than the corresponding hypothetical expectations due to the exclusion of these ‘passive’ socioeconomic functions from the calculation of entropy, where entropy values were correlated with the unemployment rate, as if the unemployed were something external to the socio-economic structure itself.

There have been no studies in the literature on the entropy of unemployment duration in relation to labour market indicators. In the presented article, crisis periods influenced the emergence of outlier entropy values in the initial phase of these crises: high during the financial crisis and low in the first year of the pandemic. Therefore, the emergence of outlier values is a regularity in this case. Only a comparison of these results with unemployment rates in the analysed years leads to an explanation of these results. The unemployment rate at the beginning of the financial crisis was high, while in the first year of the pandemic, it was low. This shows that entropy was more related to unemployment rates than to the fact that crises broke out.

## 6. Conclusions

The conducted statistical analysis allowed to achieve the research objective and to formulate answers to the research questions. When determining the past entropy for a short duration, it assumed negative values. Therefore, it is not a well-defined measure. It follows that not all the models considered correctly determine the entropy of unemployment duration. The residual entropy values allowed us to separate four clusters from the analysed period. Two of them—the years 2007–2008 (Cluster 1) and 2020 (Cluster 4) are the beginnings of two crises: the financial one and the COVID-19 pandemic, respectively. In these two cases, the entropy assumed extreme values: the highest value for Cluster 1 and the lowest for Cluster 4. In the case of the entropy of unemployment duration, there was no close relationship between entropy and the generally understood crisis situation in the labour market.

The comparison of entropy values with unemployment rates allowed answering research question Q2. This study showed a similarity between the formation of entropy of unemployment duration and the registered unemployment rate. In the analysed period, high unemployment rate values corresponded to high entropy values. This shows that in the period of high unemployment, unemployment duration distributions are less informative than in the period of low unemployment (measured by the unemployment rate). In this sense, high unemployment rates in the labour market are a destabilising element of this market, more than crisis situations.

The main limitation of this study is the access to individual data. Data collected in the Central Statistical Office in Poland are aggregated. The only source of reliable individual data on unemployment in Poland is Poviat Labour Offices. However, these data are not disseminated via the Internet platform. It can be available as anonymous data for scientific purposes. The second limitation is the fact that there are data on registered unemployment. Since some people do not use the office’s services when looking for a job, information on unemployed people from district labour offices does not fully reflect the situation in the labour market in Poland.

The conducted study may have practical application. High unemployment rates in the labour market may cause analyses related to the duration of unemployment based on survival analysis methods to have a lower information load. These methods have greater information value when the unemployment rate is lower. In such situations, the labour market is more orderly (in the sense of entropy), and events such as unemployed people undertaking work are less chaotic. The labour market, as a system, is characterised by less uncertainty.

## Figures and Tables

**Table 1:** Number of persons de-registered from the Poviat Labour Office in Szczecin, average duration of registration, and registered unemployment rate in Poland in 2007–2021.

Years	Number of De-Registered Persons		Average Duration of Registration (Months)		Registered Unemployment Rate in Poland (%)
	Total	for Work	Total	for Work	
2007	23,745	8185	12.5	13.8	11.2
2008	17,232	5434	11.7	9.8	9.5
2009	19,398	6992	4.9	4.2	12.1
2010	17,613	7259	6.1	6.3	12.4
2011	15,194	5950	7.4	7. 6	12.5
2012	15,570	6020	7.9	7.7	13.4
2013	23,762	10,979	8.5	7.5	13.4
2014	24,443	10,956	8.9	8.0	11.4
2015	25,568	11,019	8.4	7.4	9.7
2016	23,447	9897	8.4	6.6	8.2
2017	19,697	7888	7.0	5.6	6.6
2018	14,873	6180	5.7	4.8	5.8
2019	12,680	5636	5.3	4.1	5.2
2020	7772	5383	5.4	4.4	6.3
2021	8878	5621	7.7	6.3	5.8

**Table 2:** Results of the estimation of the parameter λ for exponential distribution of duration in unemployment and the values of the Shannon entropy, residual entropy and CRE.

Years	Parameter *λ*	Shannon Entropy/ Residual Entropy	CRE
2007	0.0205	4.8867	48.7489
2008	0.0271	4.6096	36.9525
2009	0.0729	3.6186	13.7169
2010	0.0672	3.6994	14.8704
2011	0.0528	3.9405	18.9253
2012	0.0492	4.0127	20.3431
2013	0.0541	3.9176	18.4971
2014	0.0505	3.9854	19.7953
2015	0.0512	3.9714	19.5200
2016	0.0504	3.9885	19.8554
2017	0.0570	3.8652	17.5523
2018	0.0726	3.6225	13.7695
2019	0.0848	3.4671	11.7878
2020	0.1283	3.0532	7.7926
2021	0.0827	3.4923	12.0895

**Table 3:** DTW distances and median and mean shifts (in years) between the entropy and unemployment rate.

Types of Entropy	Registered Unemployment Rate	Previous Year’s Unemployment Rate
DTW distances		
Shannon entropy	12.325	9.898
CRE	14.034	10.328
Median shifts		
Shannon entropy	1	0
CRE	1	1
Mean shifts		
Shannon entropy	0.700	0.000
CRE	0.792	0.440

## Data Availability

Data referring to the unemployment rate come from the Local Data Bank (Statistics Poland) (https://bdl.stat.gov.pl/bdl/start (accessed on 13 March 2025)). The dataset on the registered unemployed used in this article is not readily available because the data are a part of an ongoing study. Requests to access the datasets should be directed to the Poviat Labour Office in Szczecin (Poland).
